# ESMO 2021—highlights in colorectal cancer

**DOI:** 10.1007/s12254-022-00808-7

**Published:** 2022-04-28

**Authors:** Lukas Weiss

**Affiliations:** IIIrd Medical Department, Paracelsus Medical University, Muellner Hauptstr. 48, 5020 Salzburg, Austria

**Keywords:** Treatment, Update, KRASG12C, SIRT, COVID-19

## Abstract

This short review reflects on a personal selection of three abstracts on colorectal cancer (CRC) presented at the 2021 ESMO Congress: (1) KRASG12C as a new therapeutic target in metastatic CRC, supported by data from the KRYSTAL‑1 and CodeBreaK101 trials, (2) positive phase 3 data on the possible role of selective internal radiotherapy (SIRT) in the second-line treatment of liver-limited metastatic CRC, and (3) the impact of the coronavirus disease 2019 (COVID-19) pandemic on CRC screening, management and mortality, now and in the upcoming years*.*

## Targeted therapy in KRAS G12C-mutated metastatic CRC

Although KRAS is one of the most frequently mutated genes in colorectal cancer (CRC), only 3–4% of all patients with metastatic CRC exhibit a KRAS G12C mutation [1]. KRAS G12C is a point mutation in the KRAS gene resulting in a glycine-to-cysteine amino acid substitution at codon 12, thereby, leading to constitutive activation and oncogenesis. These patients show a worse prognosis when compared to patients with non-KRAS G12C mutated disease.

Adagrasib is a covalent inhibitor of KRAS G12C which irreversibly and selectively binds to KRAS G12C [2]. In the KRYSTAL‑1 phase 1/2 trial, adagrasib was investigated as monotherapy (*n* = 46) or in combination with the anti-EGFR antibody cetuximab (*n* = 32) in heavily pretreated KRAS G12C-mutated metastatic CRC [3]. This combination is based on the rationale that EGFR signaling has been identified as the dominant mechanism of CRC resistance to KRAS G12C inhibitors [4].

Adagrasib alone resulted in an overall response rate (ORR) of 22% and disease control rate (DCR) of 87% among 45 evaluable patients. Median progression-free survival (PFS) for monotherapy was 5.6 months (95% confidence interval [CI] 4.1–8.3). The addition of cetuximab could increase clinical efficacy to an ORR of 43% and DCR of 100% among 28 evaluable patients. Here, median time to response was 1.3 months and 71% of patients remained on treatment at the time of analysis. Grade 3/4 adverse events for combination therapy could be observed in 16% of patients, with diarrhea, acneiform rash, stomatitis and QTc prolongation being the most frequent (each 3%).

Based on these results, adagrasib and cetuximab is compared to standard chemotherapy plus/minus antiangiogenic agent in the KRYSTAL-10 trial, a phase 3 randomized trial in patients with KRAS G12C mutated metastatic CRC who have progressed after first-line treatment (NCT04793958).

These data are supported by another trial: in the phase Ib CodeBreaK101 trial, sotorasib, another KRAS G12C inhibitor, was investigated in combination with the anti-EGFR antibody panitumumab in 31 chemorefractory patients [5]. The investigators reported a confirmed plus unconfirmed ORR of 27% and a DCR of 81%.

Sotorasib is already approved by the European Medicines Agency for the treatment of patients with advanced non-small cell lung cancer whose tumors harbor a KRAS G12C mutation and who have progressed after at least one prior line of systemic therapy [6].

The combination of sotorasib and panitumumab will be investigated in the CodeBreak300 trial, a phase 3 randomized trial in patients with KRAS G12C mutated metastatic CRC in the third-line setting (NCT04793958).

## First positive phase 3 trial for selective internal radiotherapy in CRC

Selective internal radiotherapy (SIRT) or radioembolization describes the transarterial delivery of microscopic glass beads containing radioactive yttrium (Y-90) to liver metastases through hepatic tumor-feeding arteries.

The EPOCH trial investigated the role of SIRT when added to standard second-line chemotherapy in patients with metastatic CRC limited to the liver [7].

In this phase 3 study, 428 patients were randomized to either chemotherapy alone or the combination with SIRT applied in a single setting, before or after the first cycle of chemotherapy. Both primary endpoints were met, with a slight increase in median PFS (8.0 vs 7.2 months, hazard ratio [HR] 0.69; 95% CI, 0.54–0.88; *p* = 0.0013) as well as in median hepatic PFS (9.1 vs 7.2 months, HR 0.59, *p* = 0.0019) when adding SIRT. Moreover, ORR was also higher in the combination arm with 34.0% compared to 21.1% with chemotherapy alone. However, this did not translate into an increased median overall survival (14.0 vs 14.4 months, HR 1.07, *p* = 0.7229).

Grade 3 adverse events—especially neutropenia—were reported more frequently with SIRT (68.4% vs 49.3%) but not leading to dose reductions in chemotherapy.

So after failing to show an improvement in PFS or OS in the first-line setting [8], adding SIRT to standard chemotherapy to liver-limited metastatic CRC may be beneficial in the second-line setting.

## Impact of the COVID-19 pandemic on CRC screening and diagnosis

Due to the immense challenges posed by the coronavirus disease 2019 (COVID-19) pandemic to health care systems worldwide, cancer screening programs had to be scaled back or were not sought by patients out of fear of COVID-19 infection.

At ESMO 2021, Tehfe M et al. presented their analysis of data of the Canadian province of Quebec regarding fecal occult blood test and colonoscopy for CRC screening, as well as CRC surgery [9]. When comparing the 4‑month period during the first wave of the pandemic (April–July 2020) to the same time period in the preceding year (April–July 2019), the researchers could observe a dramatic drop in CRC screening but also CRC surgeries (Table 1).

**Table 1:** Drop in screening, diagnostics and surgery of CRC during COVID-19 pandemic, Province of Quebec, Canada

Intervention	Frequency (2020 vs. 2019) [%]
Fecal occult blood test	−67.26
Colonoscopy	−57.8
Surgery	−29.5

Although prior to the second wave (August–October 2020) many health care services could be resumed, CRC screening was still less than in the previous year (fecal occult blood test: −5%, colonoscopy: −11.4%) as were CRC surgeries (−28%).

These data are in line with other reports [10] and exemplify how the COVID-19 pandemic is impacting CRC management. Since CRC survival is closely linked to stage of disease [11], the delays in diagnosis of CRC are expected to lead to a stage-shift at first diagnosis as well as in an increase in emergency admissions, both known to negatively affect prognosis in CRC [12].
